# Anxiety, depression, and quality of life in postoperative non-small cell lung cancer patients under the intervention of cognitive-behavioral stress management

**DOI:** 10.3389/fpsyg.2023.1138070

**Published:** 2023-05-31

**Authors:** Fengju Wang, Shuyan Zhang, Bingbing Song, Yuxiang Han

**Affiliations:** ^1^Department of Thoracic Surgery, Harbin Medical University Cancer Hospital, Harbin, China; ^2^Department of Urology, Harbin Medical University Cancer Hospital, Harbin, China; ^3^Department of Pain Medicine, Harbin Medical University Cancer Hospital, Harbin, China

**Keywords:** non-small cell lung cancer, cognitive behavioral therapy, anxiety, depression, quality of life

## Abstract

**Objective:**

Cognitive-behavioral stress management (CBSM) is a psychotherapy that helps patients cognize and manage stress to improve mental health and quality of life. This study aimed to explore the influence of CBSM on anxiety, depression, and quality of life in non-small cell lung cancer (NSCLC) patients.

**Methods:**

In total, 172 NSCLC patients who received tumor resection were randomized 1:1 into the usual care (UC) group (*N* = 86) and CBSM group (*N* = 86) to receive 10-week UC and CBSM interventions. Moreover, all participants attended a 6-month follow-up.

**Results:**

Hospital Anxiety and Depression Scales (HADS)-anxiety score at 3^rd^ month (M3) (*P* = 0.015) and 6^th^ month (M6) (*P* = 0.018), HADS-depression score at M3 (*P* = 0.040) and M6 (*P* = 0.028), and depression rate at M6 (*P* = 0.035) were descended in CBSM group compared to UC group. Besides, depression severity was reduced at M6 (*P* = 0.041) in CBSM group compared to UC group, but anxiety severity only showed a decreased trend (*P* = 0.051). Additionally, Quality of Life Questionnaire-Core 30 (QLQ-C30) global health status score and QLQ-C30 function score at 1^st^ month (M1), M3, and M6 were elevated (all *P* < 0.05), while QLQ-C30 symptoms score was declined at M1 (*P* = 0.031) and M3 (*P* = 0.014) in CBSM group compared to UC group. Notably, the efficacy of CBSM was impressive in patients with baseline depression or undergoing adjuvant therapy.

**Conclusion:**

CBSM is a feasible intervention that effectively improves mental health and quality of life in postoperative NSCLC patients.

## Introduction

Non-small cell lung cancer (NSCLC) is responsible for about 80–85% of lung cancer, which is one of the leading causes of cancer-related deaths around the whole world (Sung et al., 2021; Chen P. et al., 2022). Generally, the treatment methods for NSCLC include surgery, radiotherapy, chemotherapy, immunotherapy, targeted therapy, etc., which have developed to some extent in recent years (Alexander et al., 2020; Mithoowani and Febbraro, 2022). However, NSCLC patients still suffer burdens of symptoms (such as pain, dyspnea, etc.), recurrence, and economics, which all further cause negative influences on their mental health and quality of life (Vijayvergia et al., 2015; Buja et al., 2021; Bowes et al., 2022; Kroll et al., 2022; Merlo et al., 2022). Notably, 30.1–49.6% of postoperative NSCLC patients experience anxiety or depression, and 6–52% of postoperative NSCLC patients experience a decrease in quality of life, which may reduce the treatment adherence of patients and even negatively affect their clinical outcomes (Moller and Sartipy, 2012; Arrieta et al., 2013; Lowery et al., 2014; Huang et al., 2020; Iovoli et al., 2022). Therefore, it is crucial to find a feasible management to improve the mental health and quality of life in NSCLC patients.

Cognitive-behavioral stress management (CBSM) is a psychotherapy that helps patients prevent maladaptive cognitions and improve their ability to cope with stress (Hofmann et al., 2012; Francis and Kumar, 2013; Nakao et al., 2021). Unlike other common psychological interventions such as reminiscence therapy which increases happiness by recalling past memories or music therapy which improves emotions and quality of life through distraction, CBSM focuses on direct stress management of patients, which helps them establish coping strategies early on to ameliorate mental health and quality of life (Kasl-Godley and Gatz, 2000; Francis and Kumar, 2013; Chirico et al., 2020). One previous study suggests that CBSM reduces the Center for Epidemiologic Studies-Depression scale score in females with non-metastatic breast cancer (Stagl et al., 2015a). In addition, another study reveals that CBSM can effectively enhance the Functional Assessment of Cancer Therapy-General (FACT-G) score in prostate cancer patients after surgery (Penedo et al., 2004). Furthermore, one research reports that an intervention involving CBSM declines the hospital anxiety and depression scale (HADS) score and increases the Short-Form Health Surveys score in cancer survivors (Borosund et al., 2020). These studies reveal that CBSM is a beneficial intervention mode to relieve patients' mental health as well as improve their quality of life. Notably, NSCLC patients may suffer from similar or even more serious mental stress and worse quality of life than the above cancer patients due to the high incidence rate and mortality (Pitman et al., 2018). Therefore, we suppose that CBSM may also be a good fit for NSCLC patients. However, there is no study reporting the effect of CBSM on NSCLC patients.

Therefore, this study aimed at investigating the influence of CBSM on ameliorating mental health and enhancing the quality of life in postoperative NSCLC patients.

## Methods

### Patients

In this randomized, controlled research, a total of 172 NSCLC patients who received tumor resection were continuously enrolled from August 2019 to November 2021. The inclusion criteria were: (a) diagnosis with NSCLC by pathology; (b) aged more than 18 years; (c) the expected survival time over 1 year; (d) received tumor resection; (e) could complete assessments in this study. The exclusion criteria were: (a) complicated with other primary carcinomas or malignancies; (b) incapable of normal communication; (c) had a severe mental disorder or cognitive impairment. This research protocol was reviewed and approved by the Ethics Committee of Harbin Medical University Cancer Hospital, approval number [2018-164-IIT]. All patients provided written informed consent.

### Study design

The included patients were randomized into two groups after receiving baseline assessments, one group received the CBSM intervention (*n* = 86) and the other group received the usual care (UC) intervention (*n* = 86). Survival status and health information were updated weekly by the charge nurses. Patients in both groups received routine follow-up for 6 months, with the first 10 weeks of intervention or education at the rehabilitation center of our hospital. The assessment scales for this study included the HADS and quality of life questionnaire-core 30 (QLQ-C30), which patients self-completed at discharge (M0), 1^st^ month (M1), 3^rd^ month (M3), and 6^th^ month (M6) after discharge. All study procedures were approved by the Ethics Committee of Harbin Medical University Cancer Hospital.

### Data collection

Demographics, concomitant disease, disease-related information, and therapy-related information of patients were collected, which were screened from the Electronic Medical Record System, entered by one investigator, and re-checked by another investigator.

### Random assignment

Random assignment (1:1) was carried out by designated investigators who were blinded to patient information using the CLEANWEB^®^ software. Blocks size was selected as 4. After randomization, the patients were told which group they were assigned to. As a result, eighty-six patients were assigned to the CBSM group, and eighty-six patients were assigned to the UC group.

### Intervention

UC was carried out for NSCLC patients in the UC group once a week for 10 weeks by trained nurses. Usual care was conducted in a group-based form (5–6 patients per group). Each weekly group training lasted about 90 min including 60-min didactic portion and 30-min free time, in which didactic portion mainly contained health education, rehabilitation care education, and answering questions.

CBSM was applied to NSCLC patients in the CBSM group once a week for 10 weeks. CBSM was conducted in a group-based form (5-6 patients per group) by trained nurses. Each weekly group training lasted about 90 min, including a didactic portion for 60 min (the same content as the UC group plus special interventions) and relaxation training for 30 min. The additional special interventions of didactic portion contained: (a) stress management, including stress awareness, cognitive adjustment, anger management, and assertiveness establishment; (b) information associated with NSCLC: including bodily changes, fears over progression, coping with symptoms, and frustration with health care; (c) pain care: including the management of surgical pain, rehabilitation pain, and cancer-related pain. The relaxation training contained deep breathing, muscle relaxation, and meditation (Penedo et al., 2008). The study sessions were shown in Supplementary Table 1. All study procedures were approved by the Ethics Committee of Harbin Medical University Cancer Hospital.

### Evaluation

The self-assessment scale questionnaires used in this study were distributed to patients at appropriate times by their charge nurses. For all patients, the HADS and QLQ-C30 scores were evaluated at M0, M1, M3, and M6. The HADS score was conducted to evaluate anxiety and depression, and the severity was classified as no (0~7), mild (8~10), moderate (11~14), and severe (15~21) (Zigmond and Snaith, 1983). The QLQ-C30 score was performed to evaluate the quality of life, in which the dimension of QLQ-C30 included pain, functions, symptoms, fatigue, etc. (Aaronson et al., 1993). Those self-assessment score results were entered by one investigator and re-checked by another investigator.

### Statistics

The sample size calculation was performed per the hypothesis that the mean M6 QLQ-C30 global health status in the CBSM group was 75, while the mean M6 QLQ-C30 global health status in the US group was 70. The standard deviation (SD) was supposed as 10. With the significance (α) level of 0.05 and the power of 85%, the minimum sample size was 72 for each group and then adjusted to 86 considering the drop-out possibility of 20%. Comparisons between the two groups were determined by the χ^2^ test, Student *t*-test, and Wilcoxon rank-sum test. *P* < 0.05 indicated significance. SPSS v.22.0 was used for data processing and GraphPad Prism v.7.0 was used for figure plotting.

## Results

### Baseline characteristics of UC group and CBSM group

**Figure 1:**
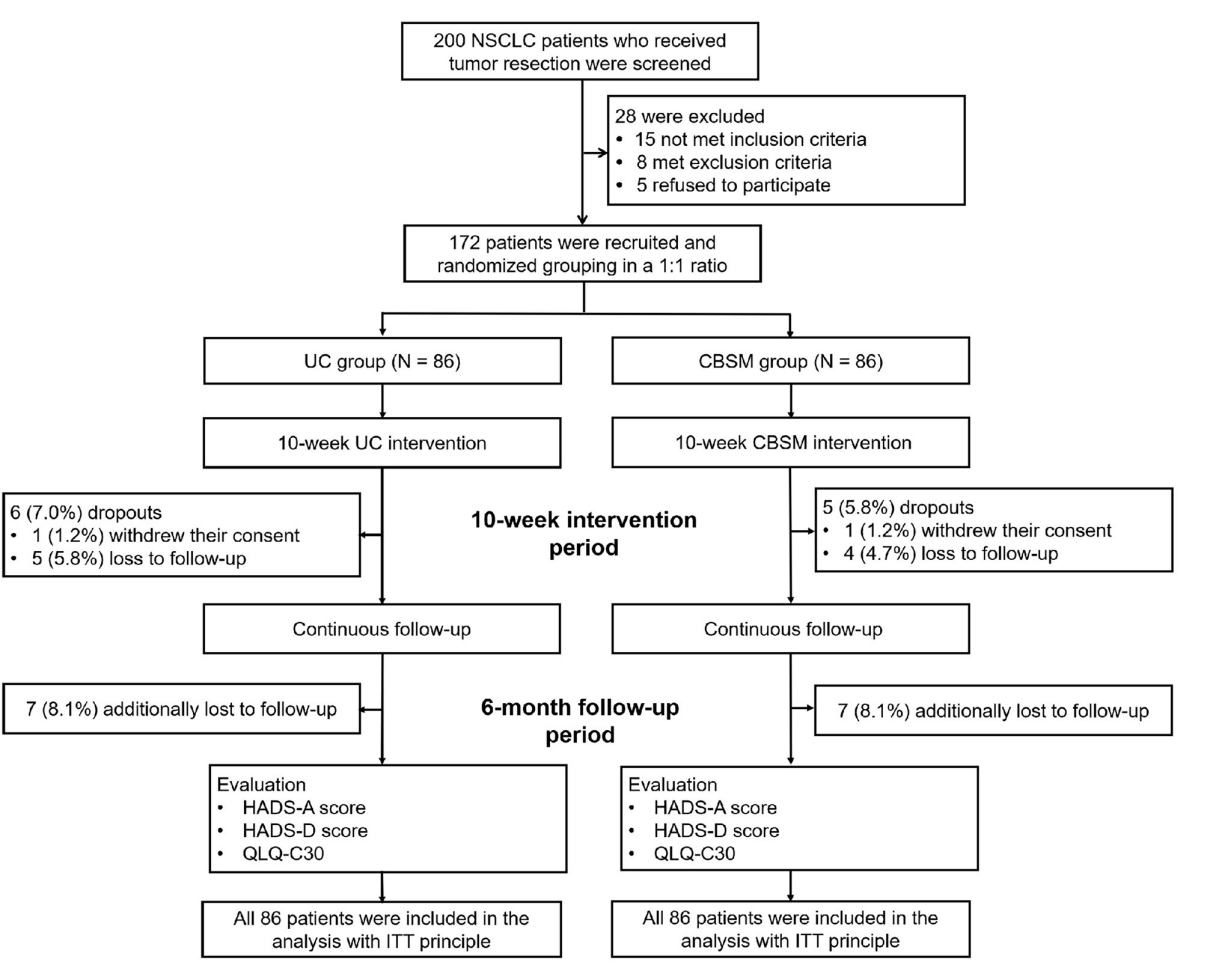
Consort diagram.

The study flow of our study was shown in the Consort Diagram (Figure 1). The UC group consisted of 66 (76.7%) males and 20 (23.3%) females, whose mean age was 59.4 ± 10.1 years. Meanwhile, the CBSM group consisted of 70 (81.4%) males and 16 (18.6%) females, whose mean age was 60.8 ± 9.3 years. Additionally, no difference was found in clinical characteristics between UC group and CBSM group, including demographics, medical histories, tumor characteristics, treatment, as well as HADS and QLQ-C30 scores at M0 (all *P* > 0.05) (Table 1).

**Table 1:** Clinical characteristics of NSCLC patients.

**Characteristics**	**UC group (*N =* 86)**	**CBSM group (*N =* 86)**	***P* value**
Age (years), mean ± SD	59.4 ± 10.1	60.8 ± 9.3	0.334^a^
Gender, No. (%)			0.453^b^
Male	66 (76.7)	70 (81.4)	
Female	20 (23.3)	16 (18.6)	
Marriage status, No. (%)			0.688^b^
Married	72 (83.7)	70 (81.4)	
Single/divorced/widowed	14 (16.3)	16 (18.6)	
Employment status before surgery, No. (%)			0.151^b^
Employed	35 (40.7)	26 (30.2)	
Unemployed	51 (59.3)	60 (69.8)	
Level of education, No. (%)			0.449^b^
Primary school or less	10 (11.6)	8 (9.3)	
High school	47 (54.7)	41 (47.7)	
Undergraduate or above	29 (33.7)	37 (43.0)	
Location, No. (%)			0.600^b^
Urban	77 (89.5)	79 (91.9)	
Rural	9 (10.5)	7 (8.1)	
History of smoke, No. (%)			0.169^b^
No	50 (58.1)	41 (47.7)	
Yes	36 (41.9)	45 (52.3)	
History of drink, No. (%)			0.429^b^
No	57 (66.3)	52 (60.5)	
Yes	29 (33.7)	34 (39.5)	
History of hypertension, No. (%)			0.349^b^
No	55 (64.0)	49 (57.0)	
Yes	31 (36.0)	37 (43.0)	
History of hyperlipidemia, No. (%)			0.101^b^
No	71 (82.6)	62 (72.1)	
Yes	15 (17.4)	24 (27.9)	
History of diabetes, No. (%)			0.688^b^
No	72 (83.7)	70 (81.4)	
Yes	14 (16.3)	16 (18.6)	
Differentiation, No. (%)			0.085^c^
Well	22 (25.6)	16 (18.6)	
Moderate	44 (51.2)	40 (46.5)	
Poor	20 (23.3)	30 (34.9)	
Tumor size >5 cm, No. (%)			0.280^b^
No	53 (61.6)	46 (53.5)	
Yes	33 (38.4)	40 (46.5)	
LYN metastasis, No. (%)			0.747^b^
No	58 (67.4)	56 (65.1)	
Yes	28 (32.6)	30 (34.9)	
Distant metastasis, No. (%)			NA
No	86 (100.0)	86 (100.0)	
Yes	0 (0.0)	0 (0.0)	
TNM stage, No. (%)			0.308^c^
1	33 (38.4)	24 (27.9)	
2	25 (29.1)	32 (37.2)	
3	28 (32.6)	30 (34.9)	
Surgery type, No. (%)			0.309^b^
Lobectomy	69 (80.2)	74 (86.0)	
Others (wedge, segmentectomy, or pneumonectomy)	17 (19.8)	12 (14.0)	
Neoadjuvant therapy, No. (%)			0.747^b^
No	56 (65.1)	58 (67.4)	
Yes	30 (34.9)	28 (32.6)	
Adjuvant therapy, No. (%)			0.439^b^
No	38 (44.2)	33 (38.4)	
Yes	48 (55.8)	53 (61.6)	
Postoperative complications, No. (%)			0.341^b^
No	52 (60.5)	58 (67.4)	
Yes	34 (39.5)	28 (32.6)	
HADS-A score at M0, mean ± SD	7.6 ± 2.6	8.0 ± 3.0	0.445^a^
HADS-D score at M0, mean ± SD	7.7 ± 3.1	8.0 ± 3.3	0.505^a^
QLQ-C30 global health status at M0, mean ± SD	61.7 ± 13.6	60.4 ± 11.5	0.488^a^
QLQ-C30 functions score at M0, mean ± SD	57.2 ± 16.5	59.2 ± 13.8	0.385^a^
QLQ-C30 symptoms score at M0, mean ± SD	36.1 ± 16.8	37.5 ± 14.8	0.571^a^

NSCLC, non-small cell lung cancer; UC, usual care; CBSM, cognitive behavioral stress management; SD, standard deviation; LYN, lymph node; NA, not available; TNM, tumor-node-metastasis; HADS-A, hospital anxiety and depression scale for anxiety; M0, at discharge; HADS-D, hospital anxiety and depression scale for depression; QLQ-C30, quality of life questionnaire-core 30. The superscripts in this table represented the type of statistical tests corresponding to the *P* values, where ^a^represented the Student *t*-test; ^b^represented the χ^2^ test; ^c^represented the Wilcoxon rank-sum test.

### Comparison of anxiety and depression between groups

**Figure 2:**
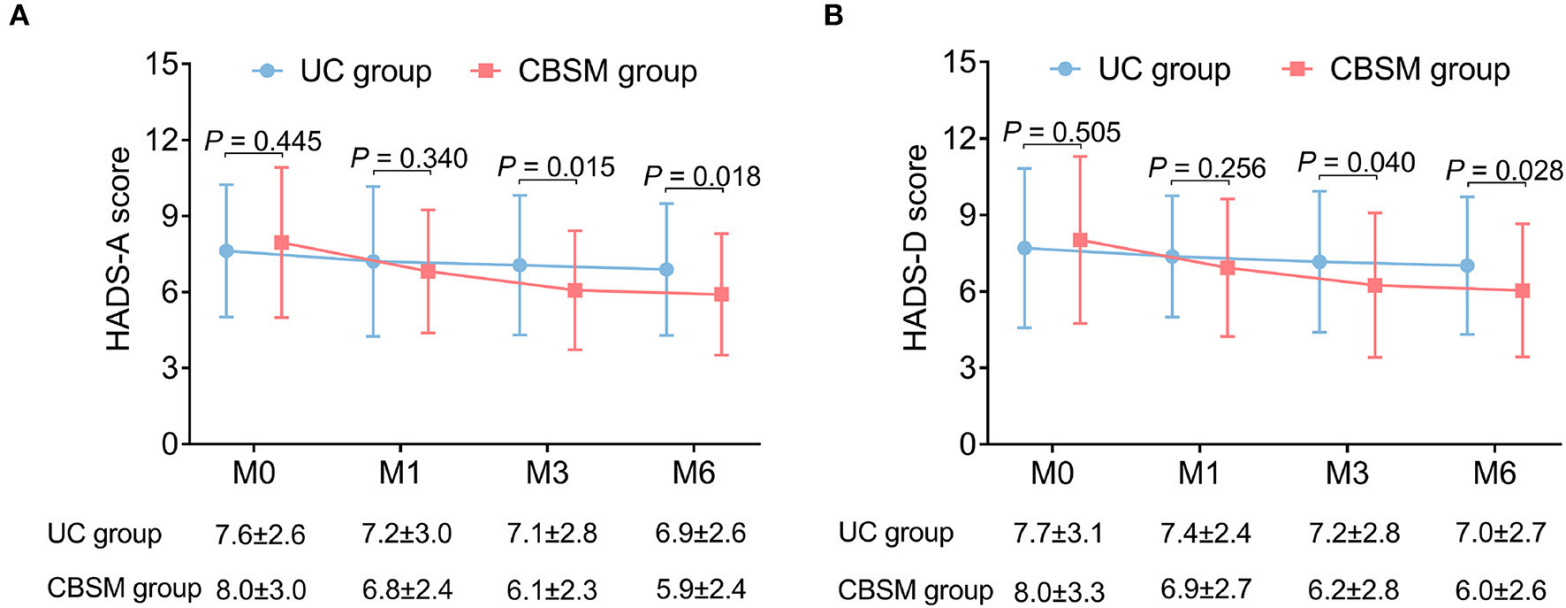
Comparison of HADS scores between groups. HADS-A score **(A)** and HADS-D score **(B)** were decreased at M3 and M6 in CBSM group compared with UC group.

HADS-A score was declined at M3 (6.1 ± 2.3 vs. 7.1 ± 2.8) (*P* = 0.015) and M6 (5.9 ± 2.4 vs. 6.9 ± 2.6) (*P* = 0.018) in CBSM group compared to UC group (Figure 2A). Besides, HADS-D score was also reduced at M3 (6.2 ± 2.8 vs. 7.2 ± 2.8) (*P* = 0.040) and M6 (6.0 ± 2.6 vs. 7.0 ± 2.7) (*P* = 0.028) in CBSM group compared to UC group (Figure 2B).

**Figure 3:**
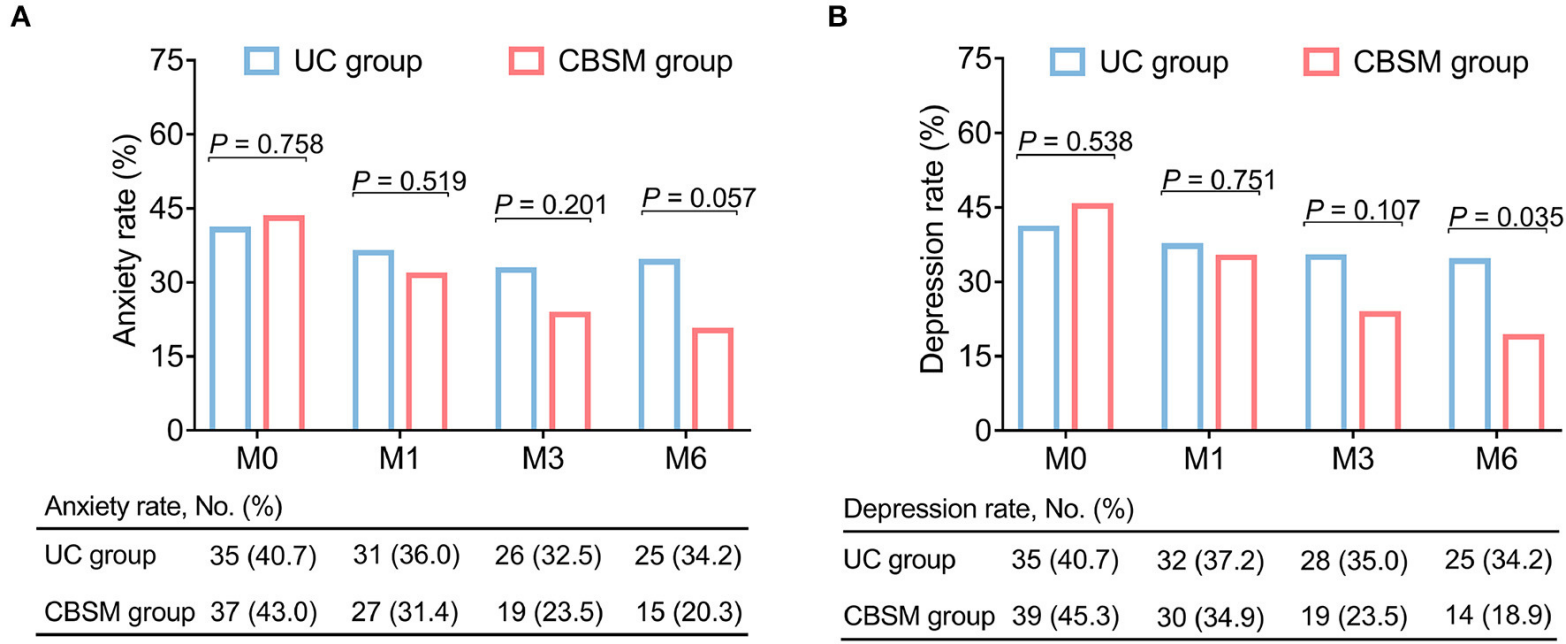
Comparison of anxiety rate and depression rate between groups. Anxiety rate showed a decreasing trend at M6 in CBSM group compared with UC group **(A)**, depression rate was reduced at M6 in CBSM group compared with UC group **(B)**.

In terms of anxiety rate, there was no discrepancy between groups at any assessment time points (all *P* > 0.05), however, anxiety rate showed a decreasing trend at M6 in CBSM group compared with UC group although it did not reach statistical significance (20.3 vs. 34.2%) (*P* = 0.057) (Figure 3A). Furthermore, depression rate was descended at M6 (18.9 vs. 34.2%) (*P* = 0.035) in CBSM group compared to UC group (Figure 3B).

In general, no discrepancy was found in anxiety severity between groups at any assessment time points (all *P* > 0.05), but anxiety severity tended to decrease at M6 in CBSM group compared to UC group without statistical significance (*P* = 0.051). Notably, depression severity declined at M6 (*P* = 0.041) in CBSM group compared with UC group (Table 2).

**Table 2:** Comparison of anxiety and depression severity among the NSCLC patients.

**Items**	**UC group, No. (%)**				**CBSM group, No. (%)**				***P* value**
	**No**	**Mild**	**Moderate**	**Severe**	**No**	**Mild**	**Moderate**	**Severe**	
**Anxiety severity**									
M0	51 (59.3)	25 (29.1)	8 (9.3)	2 (2.3)	49 (57.0)	24 (27.9)	8 (9.3)	5 (5.8)	0.626
M1	55 (64.0)	17 (19.8)	13 (15.1)	1 (1.2)	59 (68.6)	19 (22.1)	8 (9.3)	0 (0.0)	0.377
M3	54 (67.5)	17 (21.3)	8 (10.0)	1 (1.3)	62 (76.5)	16 (19.8)	3 (3.7)	0 (0.0)	0.142
M6	51 (59.3)	22 (25.6)	9 (10.5)	4 (4.7)	59 (79.7)	12 (16.2)	3 (4.1)	0 (0.0)	0.051
**Depression severity**									
M0	51 (59.3)	22 (25.6)	9 (10.5)	4 (4.7)	47 (54.7)	19 (22.1)	15 (17.4)	5 (5.8)	0.371
M1	54 (62.8)	23 (26.7)	8 (9.3)	1 (1.2)	56 (65.1)	22 (25.6)	7 (8.1)	1 (1.2)	0.738
M3	52 (65.0)	18 (22.5)	9 (11.3)	1 (1.3)	62 (76.5)	12 (14.8)	6 (7.4)	1 (1.2)	0.118
M6	48 (65.8)	17 (23.3)	8 (11.0)	0 (0.0)	60 (81.1)	9 (12.2)	5 (6.8)	0 (0.0)	0.041

NSCLC, non-small cell lung cancer; UC, usual care; CBSM, cognitive behavioral stress management; M0, at discharge; M1, 1^st^ month after discharge; M3, 3^rd^ month after discharge; M6, 6^th^ month after discharge. The HADS score was applied to evaluate anxiety and depression, with no being 0–7 scores, mild being 8–10 scores, moderate being 11–14 scores, and severe being 15–21 scores. The corresponding type of statistical test in this table was the Wilcoxon rank-sum test.

### Comparison of quality of life between groups

**Figure 4:**
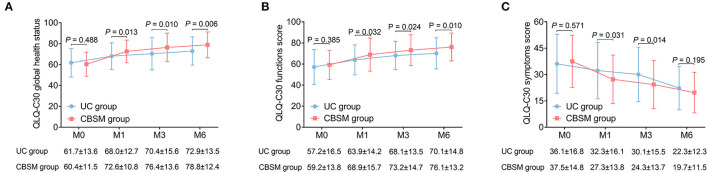
Comparison of QLQ-C30 scores between groups. QLQ-C30 global health status score was increased at M1, M3, and M6 **(A)**, QLQ-C30 function score was elevated at M1, M3, and M6 **(B)**, while QLQ-C30 symptoms score was declined at M1 and M3 **(C)** in CBSM group compared to UC group.

QLQ-C30 global health status score was increased at M1 (72.6 ± 10.8 vs. 68.0 ± 12.7) (*P* = 0.013), M3 (76.4 ± 13.6 vs. 70.4 ± 15.6) (*P* = 0.010), and M6 (78.8 ± 12.4 vs. 72.9 ± 13.5) (*P* = 0.006) in CBSM group compared to UC group (Figure 4A). Moreover, QLQ-C30 function score was ascended at M1 (68.9 ± 15.7 vs. 63.9 ± 14.2) (*P* = 0.032), M3 (73.2 ± 14.7 vs. 68.1 ± 13.5) (*P* = 0.024), and M6 (76.1 ± 13.2 vs. 70.1 ± 14.8) (*P* = 0.010) in CBSM group compared to UC group (Figure 4B). Regarding QLQ-C30 symptoms score, it was declined at M1 (27.3 ± 13.8 vs. 32.3 ± 16.1) (*P* = 0.031) and M3 (24.3 ± 13.7 vs. 30.1 ± 15.5) (*P* = 0.014) in CBSM group compared to UC group (Figure 4C).

### Subgroup analyses of outcomes at M6

In NSCLC patients without baseline anxiety, QLQ-C30 global health status was elevated at M6 in CBSM group compared to UC group (*P* = 0.010). In NSCLC patients with baseline anxiety, QLQ-C30 functions score was ascended at M6 in CBSM group compared to UC group (*P* = 0.026) (Table 3). Moreover, in NSCLC patients without baseline depression, HADS-A score was declined at M6 in CBSM group compared to UC group (*P* = 0.027). In NSCLC patients with baseline depression, HADS-D score (*P* = 0.006) was descended at M6, but QLQ-C30 global health status (*P* = 0.017) and QLQ-C30 functions score (*P* = 0.002) increased at M6 in CBSM group compared to UC group.

**Table 3:** Subgroup analysis of outcomes at M6 based on anxiety at baseline, depression at baseline, neoadjuvant therapy, and adjuvant therapy among the NSCLC patients.

**Items**	**UC group**	**CBSM group**	***P* value**
**Without anxiety at M0**			
HADS-A score at M6, mean ± SD	6.4 ± 2.4	5.5 ± 2.0	0.059
HADS-D score at M6, mean ± SD	6.6 ± 2.5	5.7 ± 2.3	0.092
QLQ-C30 global health status at M6, mean ± SD	74.8 ± 12.5	81.2 ± 10.3	0.010
QLQ-C30 functions score at M6, mean ± SD	72.7 ± 13.9	77.2 ± 12.0	0.113
QLQ-C30 symptoms score at M6, mean ± SD	21 ± 12.9	17.3 ± 9.4	0.127
**With anxiety at M0**			
HADS-A score at M6, mean ± SD	7.7 ± 2.7	6.5 ± 2.8	0.089
HADS-D score at M6, mean ± SD	7.7 ± 3.0	6.5 ± 3.0	0.115
QLQ-C30 global health status at M6, mean ± SD	69.8 ± 14.8	75.6 ± 14.3	0.135
QLQ-C30 functions score at M6, mean ± SD	65.6 ± 15.3	74.7 ± 14.8	0.026
QLQ-C30 symptoms score at M6, mean ± SD	24.4 ± 10.9	23.1 ± 13.4	0.680
**Without depression at M0**			
HADS-A score at M6, mean ± SD	6.5 ± 2.6	5.4 ± 2.0	0.027
HADS-D score at M6, mean ± SD	6.0 ± 2.1	5.7 ± 2.4	0.459
QLQ-C30 global health status at M6, mean ± SD	75.7 ± 11.8	80.2 ± 12.1	0.090
QLQ-C30 functions score at M6, mean ± SD	74.0 ± 12.5	76.2 ± 13.8	0.460
QLQ-C30 symptoms score at M6, mean ± SD	20.3 ± 12.4	18.9 ± 10.1	0.595
**With depression at M0**			
HADS-A score at M6, mean ± SD	7.4 ± 2.6	6.5 ± 2.7	0.171
HADS-D score at M6, mean ± SD	8.5 ± 2.8	6.5 ± 2.8	0.006
QLQ-C30 global health status at M6, mean ± SD	68.7 ± 15.0	77.3 ± 12.7	0.017
QLQ-C30 functions score at M6, mean ± SD	64.2 ± 16.1	76.1 ± 12.7	0.002
QLQ-C30 symptoms score at M6, mean ± SD	25.3 ± 11.6	20.6 ± 13.1	0.141
**Without neoadjuvant therapy**			
HADS-A score at M6, mean ± SD	6.4 ± 2.5	5.8 ± 2.1	0.202
HADS-D score at M6, mean ± SD	6.6 ± 2.6	5.7 ± 2.4	0.082
QLQ-C30 global health status at M6, mean ± SD	76.0 ± 12.5	81.4 ± 10.0	0.022
QLQ-C30 functions score at M6, mean ± SD	73.4 ± 14.0	77.9 ± 11.8	0.090
QLQ-C30 symptoms score at M6, mean ± SD	20.3 ± 11.9	17.8 ± 10.3	0.271
**With neoadjuvant therapy**			
HADS-A score at M6, mean ± SD	7.7 ± 2.6	6.2 ± 2.9	0.044
HADS-D score at M6, mean ± SD	7.7 ± 2.8	6.7 ± 3.0	0.224
QLQ-C30 global health status at M6, mean ± SD	67.6 ± 13.7	73.6 ± 15.3	0.149
QLQ-C30 functions score at M6, mean ± SD	64.6 ± 14.6	72.5 ± 15.4	0.066
QLQ-C30 symptoms score at M6, mean ± SD	25.6 ± 12.4	23.7 ± 13.2	0.594
**Without adjuvant therapy**			
HADS-A score at M6, mean ± SD	6.4 ± 2.6	5.5 ± 1.9	0.108
HADS-D score at M6, mean ± SD	6.5 ± 2.5	5.9 ± 2.3	0.304
QLQ-C30 global health status at M6, mean ± SD	75.9 ± 12.9	81.7 ± 11.1	0.063
QLQ-C30 functions score at M6, mean ± SD	73.3 ± 13.8	78.8 ± 13.0	0.105
QLQ-C30 symptoms score at M6, mean ± SD	19.9 ± 12.6	17.5 ± 10.5	0.404
**With adjuvant therapy**			
HADS-A score at M6, mean ± SD	7.3 ± 2.6	6.2 ± 2.6	0.051
HADS-D score at M6, mean ± SD	7.5 ± 2.8	6.2 ± 2.8	0.033
QLQ-C30 global health status at M6, mean ± SD	70.2 ± 13.6	77.0 ± 12.9	0.022
QLQ-C30 functions score at M6, mean ± SD	67.2 ± 15.2	74.4 ± 13.1	0.024
QLQ-C30 symptoms score at M6, mean ± SD	24.4 ± 11.7	21.1 ± 12.1	0.218

M6, 6^th^ month after discharge; NSCLC, non-small cell lung cancer; UC, usual care; CBSM, cognitive behavioral stress management; M0, at discharge; HADS-A, hospital anxiety and depression scale for anxiety; SD, standard deviation; HADS-D, hospital anxiety and depression scale for depression; QLQ-C30, quality of life questionnaire-core 30. The corresponding type of statistical test in this table was the Student *t*-test.

In NSCLC patients without neoadjuvant therapy, QLQ-C30 global health status (*P* = 0.022) was elevated at M6 in CBSM group compared to UC group. In NSCLC patients with neoadjuvant therapy, HADS-A score (*P* = 0.044) decreased at M6 in CBSM group compared to UC group (Table 3). In addition, in NSCLC patients without adjuvant therapy, there was no discrepancy in HADS scores or QLQ-C30 scores between groups at M6 (all *P* > 0.05). In NSCLC patients with adjuvant therapy, HADS-D score (*P* = 0.033) was descended at M6, whereas QLQ-C30 global health status (*P* = 0.022) and QLQ-C30 functions score (*P* = 0.024) were both elevated at M6 in CBSM group compared to UC group.

Additionally, by subgroup analyses based on age, gender, and TNM stage, it was revealed that the efficacy of CBSM in NSCLC patients with age <60 years, males, and NSCLC patients with TNM stage II or III was better (Supplementary Table 2).

## Discussion

Previous studies exhibit that CBSM improves anxiety and depression in many cancer patients (Bouchard et al., 2019; Tang et al., 2020). For example, one meta-analysis shows that CBSM reduces anxiety and depression in breast cancer patients (Tang et al., 2020). Moreover, another research indicates that CBSM also relieves anxiety in men with advanced prostate cancer (Bouchard et al., 2019). Similar to previous studies, our study found that CBSM decreased anxiety and depression reflected by HADS to some degree compared to UC in NSCLC patients. The possible explanations were as follows: (1) CBSM helped patients deal with negative emotions, release stress, and promoted stress management, thus alleviated anxiety and depression in patients (Nakao et al., 2021). (2) CBSM clarified the patients' knowledge of the disease, which decreased their fear of the disease and increased their confidence in the current medical level, thus relieving the patients' anxiety and depression (Chen J. et al., 2022). (3) Through relaxation training, CBSM fully alleviated the patients' physical and mental pressure, reduced their mental burden, and thus alleviated their anxiety and depression (Stagl et al., 2015b; Chen J. et al., 2022). Notably, CBSM did not reduce anxiety severity at any assessment time points and only decreased depression severity at M6 compared to UC in NSCLC patients. The possible reason was as follows: Most NSCLC patients were in the status of no/mild anxiety or depression, while the number of NSCLC patients who were in the status of moderate/severe anxiety or depression was small, resulting in a less significant effect of CBSM.

In addition to improving mental health, enhancing the quality of life in NSCLC patients is a noteworthy issue. CBSM is believed to increase the quality of life in some cancer patients (Penedo et al., 2007; Acevedo-Ibarra et al., 2019). For example, one study suggests that CBSM effectively elevates the quality of life in colorectal cancer patients (Acevedo-Ibarra et al., 2019). Another study also observes that CBSM enhances the FACT-G score in patients with postoperative prostate cancer patients (Penedo et al., 2007). Similarly, our study revealed that CBSM enhanced quality of life reflected by QLQ-C30 compared to UC in NSCLC patients. This might be because: (1) CBSM reduced the pressure and psychological burden of patients, enabling them to adjust their emotions timely and face treatment actively, thus improving their quality of life (Ghazavi et al., 2016). (2) CBSM was a skill that continuously benefited patients through 10-week interventions. Through this skill, patients could elevate their own life index in a timely and dynamic manner for continuous improvement of quality of life.

Furthermore, according to the subgroup analysis, our study found that CBSM had a better effect on patients with depression at baseline or adjuvant therapy than those without. The possible reasons were as follows: (1) Compared with the patients without depression at baseline, those with depression at baseline were more emotionally unstable. When receiving CBSM, those patients might release more pressure and negative emotions than patients without depression at baseline, so as to get a better treatment impact (Wu et al., 2021; Yu, 2021). (2) Adjuvant treatment imposed a certain burden on patients, whose side effects might negatively influence their quality of life (Fallowfield, 2005; Bezjak et al., 2008; Poghosyan et al., 2013). Therefore, patients with adjuvant therapy might benefit more from pain care and relaxation training than those without, but further exploration was needed. Meanwhile, our study also found that CBSM was more effective in patients with age <60 years, males, and patients with TNM stage II or III. These findings might be because: (1) Patients with age ≥60 years might have poor cognitive abilities and were not sensitive to CBSM care (Lovden et al., 2020). (2) The sample size of female patients in our study was relatively small. (3) Patients with TNM stage I had milder disease symptoms, better survival, and their own symptoms of anxiety and depression might be relatively mild, thus benefiting less from CBSM (Huang et al., 2020; Deng and Chen, 2023).

There were some limitations in this study: (1) Our study was a single-center study, which might lead to the bias of selection. (2) Our study only recruited postoperative NSCLC patients. While the effect of CBSM in NSCLC patients who were unable to receive surgery was unknown. (3) The assessment scale used to assess anxiety and depression was single in our study, only HADS. Therefore, future research should consider using more assessment scales. (4) It was reported that a short-term CBSM intervention would have a long-term effect on breast cancer patients (Stagl et al., 2015b). Therefore, further studies should explore the effect of long-term influence of CBSM in NSCLC patients. (5) Our study did not set blinding due to the fact that patients were easily aware of their grouping during the nursing process.

## Conclusions

In conclusion, CBSM relieves anxiety and depression, as well as improves the quality of life in postoperative NSCLC patients, particularly in those having baseline depression symptom or adjuvant therapy. Our findings indicate that CBSM may be an effective intervention for postoperative NSCLC patients. However, further multi-center studies are required for confirmation, and the effect of CBSM in NSCLC patients who are unable to receive surgery should be also investigated in future studies.

## Data availability statement

The original contributions presented in the study are included in the article/Supplementary material, further inquiries can be directed to the corresponding author.

## Ethics statement

The studies involving human participants were reviewed and approved by Harbin Medical University Cancer Hospital. The patients/participants provided their written informed consent to participate in this study.

## Author contributions

FW and YH conceived and designed the study. FW, SZ, and YH collected and analyzed the data. FW and SZ wrote the manuscript. BS and YH revised the manuscript. All authors read and approved the submitted version.
