# Supplementary material for: Anxiety, depression, and quality of life in postoperative non-small cell lung cancer patients under the intervention of cognitive-behavioral stress management
Source: Front Psychol. 2023 May 31;14:1138070. doi: 10.3389/fpsyg.2023.1138070 (PMC10264623; doi:10.3389/fpsyg.2023.1138070)
Supplement: Supplementary file 1 [file Table_1.docx]

**Supplementary Table 1**. Study sessions.

| Sessions | CBSM group | |  | UC group | |
| --- | --- | --- | --- | --- | --- |
|  | Didactic portion | Relaxation training |  | Didactic portion | Free-time |
| Phase I Trust building  (Week 1-2) | 1. Health education; 2. Encourage patients to introduce themselves and know each other; 3. Guide patients to express their current feelings; 4. Answering questions. | Deep breathing and meditation |  | 1. Health education; 2. Answering questions. | Free movement |
| Phase II Stress recognition and cognitive reconstruction  (Week 3-4) | 1. Health education; 2. Guide and encourage patients to talk about their current problems and pressures; 3. Use the specific case analysis method to introduce the mechanism of pressure and classification of pressure; 4. Answering questions. | Muscle relaxation |  | 1. Health education; 2. Answering questions. | Free movement |
| Phase III Emotion management and confidence building  (Week 5-6) | 1. Rehabilitation care education; 2. Guide patients to express their emotional changes and reasons; 3. Teach anger management and confidence management skills to help relieve patients' negative emotions; 4. Propose targeted solutions using specific case analysis methods; 5. Answering questions. | Muscle relaxation |  | 1. Rehabilitation care education; 2. Answering questions. | Free movement |
| Phase IV Information associated with NSCLC  (Week 7-8) | 1. Rehabilitation care education; 2. Invite experts to introduce relevant knowledge of diseases in the form of lectures, including management of surgical pain, rehabilitation pain, and cancer-related pain; 3. To help patients rationally overcome the fear of bodily changes and disease progression; cope with symptoms, and relieve patients' frustration with health care; 4. Answering questions. | Deep breathing and meditation |  | 1. Rehabilitation care education; 2. Answering questions. | Free movement |
| Phase V Personal summary and self-improvement  (Week 9-10) | 1. Guided patients to share the changes in their physical and mental states; 2. Answer questions and consolidate the effect of the intervention. | Deep breathing and meditation |  | 1. Review and summary; 2. Answer questions. | Free movement |

CBSM, cognitive-behavioral stress management; UC, usual care; NSCLC, non-small cell lung cancer.
