# Supplementary material for: Anxiety, depression, and quality of life in postoperative non-small cell lung cancer patients under the intervention of cognitive-behavioral stress management
Source: Front Psychol. 2023 May 31;14:1138070. doi: 10.3389/fpsyg.2023.1138070 (PMC10264623; doi:10.3389/fpsyg.2023.1138070)
Supplement: Supplementary file 2 [file Table_2.docx]

**Supplementary Table 2.** Subgroup analysis of outcome at M6 based on age, gender, and TNM stage among the NSCLC patients.

| Items | UC group | CBSM group | *P* value |
| --- | --- | --- | --- |
| **Age <60 years** |  |  |  |
| HADS-A score at M6, mean±SD | 6.9±2.4 | 5.6±2.2 | 0.009 |
| HADS-D score at M6, mean±SD | 7.1±2.8 | 6.3±2.5 | 0.193 |
| QLQ-C30 global health status at M6, mean±SD | 72.6±14.1 | 80.1±11.7 | 0.011 |
| QLQ-C30 functions score at M6, mean±SD | 68.2±14.8 | 76.9±13.4 | 0.006 |
| QLQ-C30 symptoms score at M6, mean±SD | 22.8±11.5 | 19.3±9.9 | 0.140 |
| **Age ≥60 years** |  |  |  |
| HADS-A score at M6, mean±SD | 6.9±2.9 | 6.3±2.6 | 0.410 |
| HADS-D score at M6, mean±SD | 6.9±2.5 | 5.8±2.7 | 0.090 |
| QLQ-C30 global health status at M6, mean±SD | 73.4±12.6 | 77.6±13.1 | 0.215 |
| QLQ-C30 functions score at M6, mean±SD | 73.4±14.3 | 75.3±13.1 | 0.582 |
| QLQ-C30 symptoms score at M6, mean±SD | 21.3±13.6 | 20.2±13.1 | 0.732 |
| **Male** |  |  |  |
| HADS-A score at M6, mean±SD | 6.7±2.7 | 5.6±2.2 | 0.015 |
| HADS-D score at M6, mean±SD | 7.1±2.7 | 6.0±2.5 | 0.027 |
| QLQ-C30 global health status at M6, mean±SD | 72.0±14.2 | 79.2±12.6 | 0.005 |
| QLQ-C30 functions score at M6, mean±SD | 69.8±14.8 | 76.5±13.5 | 0.011 |
| QLQ-C30 symptoms score at M6, mean±SD | 22.8±13.4 | 19.4±11.7 | 0.140 |
| **Female** |  |  |  |
| HADS-A score at M6, mean±SD | 7.4±2.4 | 7.1±2.8 | 0.757 |
| HADS-D score at M6, mean±SD | 6.9±2.9 | 6.3±3.1 | 0.596 |
| QLQ-C30 global health status at M6, mean±SD | 76.1±10.6 | 77.4±12.0 | 0.742 |
| QLQ-C30 functions score at M6, mean±SD | 71.4±15.0 | 74.5±12.1 | 0.540 |
| QLQ-C30 symptoms score at M6, mean±SD | 20.3±7.1 | 21.1±11.3 | 0.811 |
| **TNM stage I** |  |  |  |
| HADS-A score at M6, mean±SD | 6.1±2.3 | 5.5±2.0 | 0.289 |
| HADS-D score at M6, mean±SD | 6.2±2.4 | 6.1±2.6 | 0.887 |
| QLQ-C30 global health status at M6, mean±SD | 76.2±11.4 | 80.2±12.0 | 0.234 |
| QLQ-C30 functions score at M6, mean±SD | 75.1±12.6 | 75.4±13.4 | 0.935 |
| QLQ-C30 symptoms score at M6, mean±SD | 19.5±11.7 | 18.2±11.7 | 0.709 |
| **TNM stage II** |  |  |  |
| HADS-A score at M6, mean±SD | 7.3±3.1 | 5.8±2.2 | 0.059 |
| HADS-D score at M6, mean±SD | 7.6±2.9 | 5.5±2.5 | 0.010 |
| QLQ-C30 global health status at M6, mean±SD | 73.3±13.2 | 80.1±11.9 | 0.072 |
| QLQ-C30 functions score at M6, mean±SD | 69.9±16.1 | 77.8±12.4 | 0.068 |
| QLQ-C30 symptoms score at M6, mean±SD | 22.1±12.3 | 19.5±9.6 | 0.419 |
| **TNM stage III** |  |  |  |
| HADS-A score at M6, mean±SD | 7.6±2.4 | 6.4±2.9 | 0.127 |
| HADS-D score at M6, mean±SD | 7.7±2.8 | 6.6±2.8 | 0.214 |
| QLQ-C30 global health status at M6, mean±SD | 68.2±15.4 | 76.3±13.3 | 0.056 |
| QLQ-C30 functions score at M6, mean±SD | 63.5±14.3 | 74.9±14.1 | 0.008 |
| QLQ-C30 symptoms score at M6, mean±SD | 26.1±12.4 | 21.2±13.5 | 0.193 |

M6, 6th month after discharge; NSCLC, non-small cell lung cancer; UC, usual care; CBSM, cognitive behavioral stress management; M0, at discharge; HADS-A, hospital anxiety and depression scale for anxiety; SD, standard deviation; HADS-D, hospital anxiety and depression scale for depression; QLQ-C30, quality of life questionnaire-core 30; TNM, tumor-node- metastasis.

The corresponding type of statistical test in this table was the Student t-test.
